# Treatment of Tape-Worm with Elm Bark

**Published:** 1865-02

**Authors:** J. R. Dowler

**Affiliations:** Beardstown, Ill.


### TREATMENT OF TAPE-WORM WITH ELM BARK.

By J. R. DOWLER, M. D., of Beardstown, Ill.

The subject of this notice is a daughter of Mr. Eb. Fish, of Beardstown, Ill., about six years old. The only point of special interest in the case, consists in the efficiency of the remedy—to me perfectly new, and accidentally brought to my notice—which was used in its treatment.

I was treating a little brother of this patient, in the latter part of last July, a part of my prescription for whom was, as a drink, the mucilage of elm bark, made by putting pieces of the solid bark into water. The little girl was seen to be frequently eating portions of the bark during the day; the next morning, upon my visiting the boy, the mother, with much anxiety, showed me a vessel containing something that had that morning passed the little girl's bowels, with bits of the elm bark, enveloped in mucilage, which, upon examination, proved to be about three feet of tape-worm. As I supposed the passage of the worm was accidental, and had occurred simply from the looseness caused by the bark, I proceeded to prescribe what *I* supposed a much more potent anthelmintic, a large dose of turpentine and castor oil. The turpentine and oil were given several times during the three consecutive days causing pretty active purging, but with no appearance of any portion of the worm. The girl being slender, and of irritable temperament, I was forced to desist from further active medications; and partly to allay irritation of the bowels, and partly to test the influence of the bark on the worm, I directed that she should resume the use of the bark as before, by chewing and swallowing it in moderate quantities.

On visiting my *new* patient the succeeding morning, I was shown portions of the worm, mostly in separate joints, that had been passed over night. Feeling now some confidence in the anthelmintic powers of the elm back, I directed the continued use of it, in the solid form, as before, while there should be any portions of the worm passing. In my daily calls, for some days, I had the satisfaction to learn that portions of the worm continued to pass each day, and sometimes several times a day.

I now ceased to visit my patient, intending only an occasional call; but my confidence in the efficacy of *elm bark* being so well established, I advised its use to be continued, even for two or three days after any portions of the worm should be seen in the evacuations. The portions of the worm expelled, even the separate joints, were alive, showing more or less motion; a sense of their presence in the rectum, from their action, seemed to urge the patient to go to stool for their removal.

Having given direction for the links or joints to be counted care was taken by the mother to do so, and from my notes of the case, taken on the 17th of September, 1858, I find that during about seven weeks of the intervening time there had been expelled, by estimate, (taking the average lengths of the joints,) about 45 feet of the worm. At this time there had been no portions passed for two weeks, during which time the use of the bark had been omitted. The head of the worm, with about fifteen inches of the body attached, had been expelled; but thinking that all portions of the worm or *worms* might not have been removed, I advised that the patient resume the use of the bark. The next day after doing so, further portions came away, among them one about six feet in length tapering to a thread-like termination.

The next time I took notes of the case was March 23, 1859, at which time my estimate of the entire length that had been expelled footed up to 135 feet, whether of *one* or more worms I am unable to say, as in the portions I saw there were a head and tail of what I supposed one worm. Since the last estimate there have been joints occasionally evacuated.

This patient when first treated was thin in flesh—had been growing so for some two years—attended with the usual nervous symptoms, startings out of sleep, variable appetite, etc., but with no great departure from good health.

Some time during the autumn of 1863, Charles Wells, a discharged soldier, applied to me for treatment for "fits," as he called his disease, stating he had been discharged from the service on account of them. He stated that while under treatment for the "fits" by the regimental surgeon, he had discharged some small portions of tape worm, after which the surgeon treated him actively with the view of removing more of the worm, but without success.

His "fits" (epileptic) continuing to recur frequently, I

supposed they might be caused or sustained by unexpelled tape worm, and being desirous of further testing the powers of the elm bark, as an anthelmintic, I at once put him on the use of it, directing him to chew and swallow it in considerable quantities, using the brittle, thick kind of fresh bark, and directing him, in case his bowels should not be relaxed by the bark, to use small doses of oleum ricini to maintain that condition, without any restrictions as to his diet or habits of living. As he resided about eight miles from my place of residence, I requested him to report to me only occasionally.

In about five or six days, he called to see me again, stating that on the second day after commencing the use of the bark, portions of worm, mostly in separate joints, commenced coming away, and continued to do so at nearly every stool, to the time of this report of his case; he having had to use occasionally a little castor oil to promote laxity. I ordered the continuance of the same course while any portions of worm should appear in the evacuations.

Two weeks from his first report, he called again, bringing with him about seven feet of worm, tapering in width from about half-an-inch to a mere thread-like termination. He also stated that there had been daily evacuations of portions of worm, in short pieces, but mostly in separate joints, frequently passing off involuntarily, while walking about, as it appeared by the accumulation of the joints, with the elm mucilage, within the anus. This was the last report he made of his case, as after this he passed beyond my range of observation.

The foregoing, I think, must be accepted as proof that this bland agent possesses properties that make it a very efficient tape worm expelling substance.

As to the influence of this very bland agent in the dislodgement of the tape-worm, in these cases I think there can be no doubt, whatever may be the *theory* of its action.

The mode by which this parasite maintains its position must be by its verminous contraction, and adaptation of its flat surface to the inner surface of the bowels, thus preserving itself from being carried along with their fœcal contents and cast off. That it should maintain its position by the force of *suction*, exerted by the mouth, as some writers have supposed, would seem to be absurd, when the great length of the animal is considered, and the forces in operation tending to its removal. The passage of portions of the worm so promptly on the use of the bark, and the ceasing to do so on its discontinuance—even while active anthelmintics were used—leave no room to doubt its effectiveness in this case, at least, as a worm expelling agent.

It seems probable that the bark, with its thick mucilage, so interposes between the animal and the inner surface of the bowels as to prevent its lateral grasp on their surface, in consequence of which it is compelled to yield to the forces naturally operating, and is carried out with the discharges. But, as my object was simply to state the practical *facts* in the cases, I will offer no further reflections.

